# Endogenous human herpesviruses 6A/B

**DOI:** 10.1128/jvi.01054-25

**Published:** 2025-08-01

**Authors:** Louis Flamand, Jesse Arbuckle, Pascale Bonnafous, Vincent Descamps, Joshua A. Hill, Ruth Jarrett, Keith Jerome, Benedikt B. Kaufer, Rie Koide, Anthony L. Komaroff, Peter Medveczky, Hiroki Miura, Yasuko Mori, Nicholas Parrish, Philip E. Pellett, Michael Wood, Tetsushi Yoshikawa, Danielle M. Zerr

**Affiliations:** 1 Department of Microbiology, Infectious Disease and Immunology, Faculty of Medicine, Université Laval 4440, Québec, Canada; 2 Axe Maladies Infectieuses et Immunitaires, Centre de Recherche du CHU de Québec, Université Laval 4440, Québec, Canada; 3 Molecular Genetics Section, National Institute of Allergy and Infectious iseases, National Institutes of Health 2511, Bethesda, Maryland, USA; 4 Laboratoire de Virologie, Sorbonne Université, INSERM, Institut Pierre Louis d'Epidémiologie et de Santé Publique, Assistance Publique-Hôpitaux de Paris (AP-HP), Hôpitaux Universitaires Pitié-Salpêtrière Charles-Foix 27063, Paris, France; 5 Service de dermatologie, Hôpital Bichat, AP-HP, Paris, France; 6 Vaccine and Infectious Disease Division, Fred Hutchinson Cancer Center 7286, Seattle, Washington, USA; 7 MRC-University of Glasgow Center for Virus Research, Glasgow, United Kingdom; 8 Institute of Virology, Freie Universität Berlin 9166, Berlin, Germany; 9 Genome Immunobiology RIKEN Hakubi Research Team, RIKEN Cluster for Pioneering Research and RIKEN Center for Integrative Medical Sciences 198286, Yokohama, Japan; 10 Division of General Medicine, Department of Medicine, Brigham and Women's Hospital, Harvard Medical School 1811 , Boston, Massachusetts, USA; 11 College of Medicine, University of South Florida 7831, Tampa, Florida, USA; 12 Department of Pediatrics, Fujita Health University School of Medicine 89305, Toyoake, Japan; 13 Division of Clinical Virology, Center for Infectious Diseases, Kobe University Graduate School of Medicine 38303 , Kobe, Hyogo, Japan; 14 Department of Surgery, Duke University School of Medicine 12277 , Durham, North Carolina, USA; 15 Wayne State University 2954, Detroit, Michigan, USA; 16 Seattle Children’s Research Institute, Seattle, Washington, USA; 17 Division of Infectious Diseases, Department of Pediatrics, University of Washington 7284, Seattle, Washington, USA; The University of Arizona, Tucson, Arizona, USA

**Keywords:** eHHV-6, iciHHV-6, endogenous HHV-6, inherited chromosomally integrated HHV-6, human herpesvirus 6, HHV-6

## Abstract

Human herpesviruses 6A and 6B (HHV-6A/B) can integrate into the germline, resulting in inherited viral DNA—now proposed to be called “endogenous HHV-6A/B (eHHV-6A/B).” Present in 0.2–3% of humans, this integrated DNA is passed to offspring and may reactivate, posing health risks such as angina or lupus. To reduce confusion caused by varied terminology, researchers advocate using “eHHV-6A/B” for inherited forms and reserving “chromosomally integrated” for somatic integrations only.

## COMMENTARY

Most humans are infected with human herpesviruses 6B and/or 6A (HHV-6B/A) and are at risk of reactivation of these viruses, especially when immunocompromised (1–3). On numerous occasions in human (and possibly hominin [4]) history, HHV-6A/B integrated their genomes into the host germline. Therefore, the virally derived DNA has been passed on to descendants in a Mendelian manner and is present in the chromosomes of every diploid cell.

The viral genomic DNA integrates into the telomeric region of chromosomes and is present in the same chromosomal location in members of the same family (5–9). This inherited HHV-6A/B DNA is somewhat analogous to the endogenous retroviral DNA that constitutes about 8% of the human genome.

Chromosomally integrated HHV-6A/B can reactivate, express viral genes, and even produce infectious progeny, as isolation of infectious virus identical in sequence to the endogenous HHV-6 has been documented (10–13). Viral DNA integration represents one way to achieve latency: the conventional means of achieving latency in herpesviruses, episomal viral DNA, has not yet been demonstrated in HHV-6A/B.

Depending on ancestry and geographic region, the prevalence of humans carrying endogenous HHV-6A/B varies between 0.2 and 3% (14–18). The related health consequences of carrying inherited HHV-6A/B, and the implications for medical practice, are the subject of active investigation (19). Preliminary evidence suggests that the condition may increase the risk for angina pectoris (17, 20), preeclampsia (21), and systemic lupus erythematosus (18).

Scientists have used several names for this condition, including “chromosomally integrated HHV-6 (or ciHHV-6),” “inherited chromosomally integrated HHV-6 (or iciHHV-6),” or, more recently, “endogenous HHV-6 (eHHV-6)” (4, 18). This has created confusion in the literature.

To avoid confusion, the undersigned authors who study HHV-6A/B urge that all investigators adopt the term “eHHV-6A/B” when referring to inherited HHV-6A/B DNA. For the next few years, after using eHHV-6A/B for the first time in a manuscript, authors should add a parenthetical “(previously called ciHHV-6 or iciHHV-6),” for clarification. As virologists and many health care providers and biomedical scientists are familiar with the term “endogenous retroviral DNA,” the use of the term eHHV-6A/B is simpler, shorter, and also clearer.

Similarly, the term “ciHHV-6” should be used only to refer to *somatic* chromosomal integrations.

A draft of this letter was posted on the HHV-6 Foundation website, inviting scientists to join in signing this letter (22).

The undersigned commit ourselves to using this terminology exclusively, henceforth.
